# “Dressed like boys, hair trimmed, a *nalla kutti* otherwise”: construction of queer suicide in Indian online news media

**DOI:** 10.3389/fsoc.2024.1370517

**Published:** 2024-05-24

**Authors:** Khuman Bhagirath Jetubhai

**Affiliations:** Thapar Institute of Engineering and Technology, Patiala, India

**Keywords:** mass media, news reporting, LGBTQ+, journalist, sexuality, suicide

## Abstract

Suicide is a significant newsworthy event, and the media often cover cases involving queer individuals. However, there is a notable lack of research on the quality of reporting of queer suicide cases within the Indian context. This article aims to address the existing gap in Indian online news media by investigating the portrayal of queer suicide via content analysis. Content analysis involves qualitatively condensing and interpreting data to extract key consistencies and meanings from a plethora of qualitative material. The newspapers considered span from 2005 to 2022, with data collection conducted in 2023. The author alone identified news articles on queer suicide and conducted the subsequent content analysis. The study reveals that reporting on queer suicide tends to divide queer couples into the gender binary and describes what it deems to be careless sexual conduct driven by obsessive queer love, which, in turn, is blamed for the suicide. Moreover, these reports often do the following: feature families who refuse to accept their children’s identities, adopt dread-filled tones, and cite experts who provide incorrect information while engaging in victim blaming. As a result, the quality of queer suicide reporting in Indian newspapers is deemed substandard and offensive. To address this issue, the study proposes the need for training and curriculum updates in journalistic courses. This way, reporters can develop the skills necessary to sensitively and respectfully report on queer individuals in general and on queer suicide in particular.

## 1 Introduction

The phenomenon of suicide being perceived as “newsworthy” (Armstrong et al., 2020, p. 1; Kar et al., 2021, p. 2) combined with the increasing media presence of queer^1^ sexuality (Wishbox Studio, 2023)—a topic that has consistently captured readers’ attention and imagination while sparking debates due to its taboo nature (Thomson, 2022)—has led media outlets to utilise queer suicide reporting to their advantage. Ghosh (2022, p. 3) asserts that queer suicide not only has the potential to generate news but also sustains viewer interest (Johns et al., 2019, 2020).

In general, suicide poses a significant challenge to public health (Beam et al., 2018, p. 15). In the context of India, studying suicide is imperative given the country’s alarming suicide rates, estimated to range between 18 and 21 deaths per 100,000 population, surpassing the global average of 11 deaths per 100,000 population (World Health Organization, 2014; India State-Level Disease Burden Initiative Suicide Collaborators, 2018). This translates into an estimated 230,000–250,000 suicide-related deaths annually (Armstrong et al., 2020, p. 1). Of particular concern is the issue of queer suicide, as it is well-established that younger non-heterosexual individuals face an elevated risk of suicidal behaviour—a concern recognised for approximately two decades (Cover, 2012b, p. 1,173). Studies indicate that young individuals identifying as LGBTQ+ are over four times more likely to attempt suicide than their heterosexual counterparts (Johns et al., 2019, 2020).

This study differs from earlier ones conducted in India by excluding psychological and sociological perspectives and instead incorporating journalistic discourse, which significantly contributes to the understanding of psychological and sociological issues. Journalists, as rhetoricians, practice “people production or meaning work to define and narrate characters or in particular ways for particular goals” (Pullen, 2024, p. 89). Thus, what and how they choose to cover matters (Lonsdale, 2021, p. 135). Appropriate suicide reporting is essential because it allows journalists to help people understand underlying social issues, prevent further tragedies, and raise awareness of mental wellness in the community (Canadian Journalism Forum on Violence and Trauma, 2020, p. 34). Conversely, inappropriate reporting might lead to adverse effects, exemplified by the phenomenon of copycat suicide.^2^

While media portrayals of queer suicide often commodify queer identities to attract more readers and viewers, they remain insightful for researchers (Cover, 2012a, p. 20). Therefore, this research aims to analyse media content related to queer suicide. To achieve this goal, the research focuses on internet news reports of queer suicides. In the internet era, online news reports have become highly accessible, with statistical evidence suggesting that online suicide stories attract many views (Armstrong et al., 2020, p. 14) and can become viral through easy dissemination on social media and other internet channels (Kar et al., 2022c, p. 2). Given India’s expanding internet access and the generation shift towards digital news consumption (Aneez et al., 2019), studying online news media reports becomes imperative.

Understanding the public’s perception of LGBTQ+ suicide is essential (Wolff et al., 2014, p. 4). News reports and popular cultural products such as cinema and books are valuable mediums to achieve this aim. This research undertakes a content analysis of online news media reports in India, in both English and Hindi, regarding incidents of queer suicide. The study covers the period from 2005 (which marks the inception of online newspaper publications) to 2022 (the time of this research).

This paper employs queer theory, which provides a critical framework to analyse the operations by which power solidifies and validates particular forms and representations of sexuality and gender. It also examines how others are labelled as abnormal (Ruhsam, 2017). Among the most prominent scholars in this area are Michel Foucault, Gayle Rubin, Eve Kosofsky Sedgwick, Judith Butler, and Teresa de Lauretis. Queer theory is concerned with the complicated connections that influence how gender and sexual identity are understood and expressed within politics, culture, law, and history. It also examines the efforts of dominant forces to unify gender and sexual identities, and how these have affected minority identities. Queer theory focuses on the ways in which gender and sexuality as social constructions regulate gender and sexual identities, thus contributing to public discourses about sexual orientation and gender identity.

With queer theory as its basis, this paper examines the forms of marginalisation and delegitimisation that people with non-heterosexual sexualities and gender identities differing from binary norms experience through news reports’ narratives. Suicide is often attributed to the perceived illegitimacy of their sexual expressions or non-conforming gender identities. These narratives portray queer individuals negatively, depicting them as senseless and immoral, ostensibly leading themselves towards self-destruction through careless and uninhibited sexual behaviour. Such a narrative seeks to serve as a guide for readers, instructing them on what behaviours to avoid in order to prevent a life of misery. It suggests that normativity is the path to happiness and peace. This situation underscores the prevalence of the heterosexist narrative that journalists perpetuate. The narrative is accompanied by an implicit threat, i.e., that deviating from it might result in agony or, worse, the end of one’s life.

The primary focus of this paper lies in the analysis of news reports, a fundamental component of the cultural landscape, to explore the prevailing narratives through which suicide reports portray the deaths of queer individuals in India. Through this exploration, the research sheds light on the efforts that institutions, specifically media houses, make in their news reports to either normalise or stigmatise gender and sexual identities and desires. Additionally, this paper offers insight into the mechanisms through which these identities are regulated, involving various parties such as journalists responsible for crafting the news reports as well as the parents, coworkers, relatives, neighbours, teachers, and classmates of queer individuals, who are quoted in these reports.

The implications of this research extend to the media industry, social workers, mental health practitioners, and researchers involved in framing suicide prevention guidelines and public policies as well as future funding decisions. Furthermore, this study is essential for advancing research on queer mental health and suicide, particularly in India and other parts of the world. The prominent themes emerging from this research might help society comprehend how the news media either support or counter queerphobia and heterosexism—the root causes of queer social issues.

## 2 Legal and societal perspectives on queer identities in India and their portrayal in Indian media

The roots of queer identities and sexuality in India can be traced back to ancient times, as evidenced by historical writings and oral traditions dating back over 2,000 years (Pattanaik, 2015; Vanita and Kidwai, 2016). Notably, the Hijra community, encompassing transgender, intersex, and/or eunuch individuals, played significant roles in Hindu religious texts and during the Mughal era (Reddy, 2006). In a landmark decision in 2014, the Supreme Court officially recognised Hijras as the third gender (CLPR, 2018). Other indigenous sexual identities such as Kothi (sexually receptive and relatively feminine) and Panthi (the masculine insertive partners of hijra and kothi) further enrich the diverse landscape of sexuality in India (Stief, 2017). Unlike Western cultures, in which sexual identities often fit into distinct categories (i.e., heterosexual, homosexual, or bisexual), India embraces fluidity, with sexual roles and behaviours transcending conventional labels (Khan, 2001; Patel et al., 2012).

The colonial imposition of Section 377 in 1861 criminalised same-sex love, marking a shift towards intolerance in India (Vanita, 2013). However, the repeal of Section 377 in 2018 was a significant step towards the recognition of queer rights, allowing consensual same-sex acts in private (Navtej Singh Johar v. Union of India, 2018). Yet, the recent refusal to grant marriage rights to same-sex couples (Ellis-Petersen, 2023) underscores ongoing challenges faced by the queer community and highlights the complex journey from historical acceptance to colonial criminalisation and post-independence legal advancements.

The impact of anti-queer legislation on societal attitudes has been documented in various studies. For instance, Rao et al. (2020) underscored how laws such as Section 377 perpetuated social and legal discrimination against LGBTQ+ individuals, exacerbating the stigma they endured. Conversely, the decriminalisation of Section 377 marked a significant milestone in acknowledging and embracing homosexual individuals within Indian society (Singpho, 2022), a sentiment echoed by Kaur and Stephen (2019) who noted its positive effects on the homosexual community.

Nevertheless, the residual cultural and psychological effects of Section 377 persist post-decriminalisation, continuing to impact India’s LGBTQ+ community negatively (Rao et al., 2020). Maji et al. (2024) found that negative attitudes towards homosexuality prevail among engineering students, highlighting persistent societal biases. Similarly, Sorathiya et al. (2023) revealed a significant proportion of medical graduate students harbouring negative attitudes towards LGBTQ+ individuals despite overall positive trends.

Jamwal (2023) observed contrasting dynamics between urban and rural areas regarding LGBTQ+ acceptance. Urban centres have witnessed a surge in LGBTQ+ individuals openly expressing their identities, accompanied by a growing acceptance, whereas rural areas often maintain secrecy due to entrenched traditional norms. Nonetheless, Jamwal noted a gradual shift in societal attitudes, particularly among the younger demographic, attributed to factors such as decriminalisation, increased media representation, advocacy efforts, and pride events (Dhabhar and Deshmukh, 2021; Jamwal, 2023).

Concerning media representation, popular culture serves as a powerful tool in shaping societal norms and ideologies (Goel, 2021). Media messages profoundly influence collective mindsets, making accurate and inclusive portrayals of queer identities crucial (Kaur, 2018). Given most people’s reliance on media platforms for information and entertainment, media representation holds significant sway (Kanwar and Singh, 2021). In the context of queer identities, media representation not only aids in the development of gender identity, self-perception, and societal acceptance, but also influences public perceptions of the queer community (Randev, 2022).

The research highlights a significant disparity in representation statistics between Indian media and Western media, as noted by Martel (2020). This limited portrayal also reveals concerning trends, as mass media often depict queer individuals in stereotypical ways, thereby impacting their lived experiences (Phogat and Verma, 2022). While Indian cinema has transitioned from caricatured depictions to more nuanced portrayals of queer characters, commercial success remains elusive, and entrenched gender biases persist in popular culture (Manukriti and Ajay, 2020; Joy et al., 2023). Efforts to bring authentic representation to mainstream discourse through cinema have been highlighted (Kaur, 2017).

Positive representations of queer characters in advertisements help empower the queer community and foster inclusivity (Chauhan and Shukla, 2016). Moreover, research indicates a shift in Indian media coverage of LGBTQ issues from a diplomatic stance to a proactive and reformist one, with most stories adopting an equality stance and a positive attitude (Parthasarathi and Kumari, 2019). In the contemporary landscape, both traditional and new media must play a bigger role in educating the public about important queer issues by shedding light on marginalised queer identities (Randev, 2022). Further research beyond cinema is advocated to gain comprehensive insights into media representations of the queer community.

## 3 Literature review

Effective and sensitive reporting on suicide is of paramount importance, as it has the potential to influence the prevalence of suicide. Stack (2003) discovered a direct correlation between increased media coverage of suicide and a rise in suicide rates. This underscores the critical need for responsible and sensitive suicide reporting. Several international studies have been conducted to identify issues and errors in reporting that can inform responsible reporting practices (Blood et al., 2007; McTernan et al., 2018; Fong, 2021; Sørensen et al., 2021; Arafat et al., 2022). These studies have shed light on the generally poor quality of suicide news reporting. Notably, they indicate that journalists in countries like the US, Australia, Iraq, Sri Lanka, Ireland, and Malaysia frequently fail to adhere to established reporting guidelines aimed at minimising potential harm resulting from irresponsible coverage of suicide (Blood et al., 2007; McTernan et al., 2018; Fong, 2021; Sørensen et al., 2021; Arafat et al., 2022).

Turning away now from general population suicide reporting to the LGBTQ+ population on an international scale, it becomes evident that media play a pivotal role in either garnering support for or perpetuating discrimination against LGBTQ+ individuals. A study conducted in Singapore reveals that language has been strategically employed to influence national and regional discussions concerning LGBTQ+ populations (Phillips, 2021). This underscores the importance of responsible reporting practices in safeguarding the rights and acceptance of LGBTQ+ communities, as well as reducing the risk of suicide among LGBTQ+ individuals resulting from irresponsible media coverage. However, the reality is quite different. Research in the US found that newspaper articles addressing issues such as gay teen suicide and gay bullying often lack explicit references to key factors like homophobia, heterosexism, and practical strategies for addressing anti-gay bullying (Greene, 2013).

Similarly, another study highlighted the fact that news reporting on LGBT youth suicide frequently fails to address the connections between heteronormativity, mental health, depression, and despair (Cover, 2012b). Paceley and Flynn’s (2012) research reveals an overrepresentation of white males in reports on queer youth bullying, while another study found that news reporting on transgender suicides in UK newspapers often does not adhere to responsible reporting standards (Bolzern et al., 2019).

Numerous studies have assessed the quality of suicide reporting in Indian newspapers in English and other regional languages. Krishnamurthy et al. (2022, p. 1) argue that the media tends to “scapegoat, simplify, speculate, and sensationalise suicide-related news” rather than guide people to seek help. Other research has found that news reporting on suicide is often brief, simplistic, and graphic, making it highly dangerous for readers (Armstrong et al., 2018, p. 6). Additional studies have highlighted this irresponsible and subpar quality of reporting (Jain and Kumar, 2016, p. 1; Menon et al., 2022b, p. 4).

Heightening the concern, multiple studies have shown that suicide reporting lacks content that sensitises and educates readers about the issue (Jain and Kumar, 2016; Menon et al., 2020, 2021, 2022a; Kar et al., 2022a,b). Reports often omit critical information such as helpline numbers, the availability of mental health support during crises (Chandra et al., 2014, p. 693; Menon et al., 2022b, p. 3), professional opinions, suicide warning signs (Kar et al., 2022b, p. 4), and suicide-related statistics (Menon et al., 2020, 2022b). In response to such insensitive and careless reporting, researchers advocate for educating and training media professionals to engage in responsible suicide reporting (Jain and Kumar, 2016; Krishnamurthy et al., 2022).

Studies have also revealed that suicide reporting often fails to adhere to WHO suicide report guidelines or Press Council of India (PCI)-prescribed guidelines (Chandra et al., 2014; Menon et al., 2020, 2021; Kar et al., 2022a). Moreover, there is a lack of systems in place to ensure strict compliance with the framed guidelines (Arafat et al., 2020, p. 21). Comparative studies have consistently shown that English reporting on suicide is of higher quality compared to its regional counterparts (Jain and Kumar, 2016; Ganesh et al., 2020; Kar et al., 2021; Menon et al., 2021, 2022b; Raj et al., 2022).

In discussing studies on queer suicide reporting in India, Ghosh (2022, p. 2) notes that, historically, queer suicide in the Indian media received less attention than it does now. Mainstream media largely ignored the matter until it began making front-page news. She objects to the prevailing media trend of sensationalising the existence of a lesbian individual, portraying such a person as a solitary spectacle with no recognition of the broader social and personal circumstances shaping her life. Ejaz and Moscowitz (2020, p. 7), while analysing news stories covering the death, under mysterious circumstances, of a gay university professor named Ramchandra Siras, found that the case essentialised gay identity, signified civil rights and citizenship, complicated sexuality and consent, problematised the public/private boundaries of sexuality, and negotiated competing claims of morality. Despite this growing media interest, only limited scholarly work has analysed the portrayal of queer suicide in the media (Kar et al., 2021).

This literature review suggests that the quality of reporting on suicide in the general population is poor and often fails to adhere to the guidelines set forth by the World Health Organization (WHO). However, from this, it is difficult to extrapolate the quality of LGBTQ+ suicide reporting, especially considering that journalists covering sexual and gender minorities are expected to possess a higher degree of sensitivity and knowledge about the community (Geertsema-Sligh et al., 2020) compared to those reporting on suicide in the general population.

As mentioned earlier, studies that focus specifically on queer suicide in the Indian context are scarce and have limited scope. In contrast, this research encompasses all suicide incidents reported from 2005 to 2022, spanning all queer identities. This paper identifies and queers the dominant frames that emerge from news reports of suicides. It asserts that the quality of queer suicide reporting is often inadequate, revealing a lack of knowledge among journalists, which might further marginalise the queer community.

## 4 Methodology

This study employs qualitative content analysis to identify prominent framing devices used in reporting on queer suicide in both English and Hindi online news stories. Qualitative content analysis, as defined by Patton, involves “qualitative data reduction and sense-making efforts” to distil core consistencies and meanings from a volume of qualitative material (Patton, 2002, p. 453). This method goes beyond word counting or objective content extraction to examine the meanings, themes, and patterns that might be explicit or hidden within a given text. It enables researchers to understand social reality subjectively yet scientifically (Zhang and Wildemuth, 2024). Previous research works have conducted quantitative content analysis on news reports (Al-Naggar and Al-Jashamy, 2011; Zafri et al., 2021; Oosthuizen, 2022). The present research maintains a similar methodology.

The process followed for the content analysis is outlined as follows:

### 4.1 Collecting suicide news reports

The primary data source for this content analysis comprises Hindi and English web news reports identified from a comprehensive list available on Wikipedia (Wikipedia Contributors, 2023).

In identifying English news reports on queer suicide cases, the following search terms were employed: “same-sex suicide,” “LGBT suicide,” “LGBTQI suicide,” “LGBTQIA suicide,” “LGBTQ+ suicide,” “gay suicide,” “homosexual suicide,” “lesbian suicide,” “transgender suicide,” “bisexual suicide,” “queer suicide,” “intersex suicide,” “hijra suicide,” “kinnar suicide,” “third gender suicide,” “asexual suicide,” and “eunuchs suicide.”

To locate Hindi news reports on queer suicide cases, the following Hindi search terms were combined with widely used Hindi synonyms for suicide: ‘जान देदी’ [committed suicide], ‘खुदकुशी’ [suicide], and ‘आत्महत्या’ [suicide]. The specific terms used for the queer community in Hindi were: ‘बाईसेक्सुअल’ [bisexual], ‘गे’ [gay], ‘किन्नर’ [transgender], ‘समलैंगिक’ [gay], ‘ट्रांसजेंडर’ [transgender], ‘उभयलिंगी’ [bisexual], ‘थर्ड जेंडर’ [third gender], ‘तृतीयपंथी’ [third gender], ‘इंटरसेक्स’ [intersex], ‘हिजड़ा’[transgender], ‘एलजीबीटी’ [LGBT], ‘एलजीबीटीक्यू’ [LGBTQ], ‘एलजीबीटीक्यूआई’ [LGBTQI], and ‘एलजीबीटीक्यूआईए’ [LGBTQIA]. In this manner, 145 queer suicide news reports published between 2005 to 2022 were found.

### 4.2 Identifying data

The collected suicide news reports were carefully studied. Problematic statements were identified, coded, and placed in a separate column in a Microsoft Excel spreadsheet.

### 4.3 Developing themes, sub-themes, and sub-sub-themes

After a comprehensive examination of the problematic statement, themes, sub-themes, and sub-sub-themes under which the data could be classified were inductively developed, as shown in Table 1.

**Table 1:** Themes, sub-themes, sub-sub-themes, and codes used in the content analysis.

Theme	Sub-theme	Sub-sub-themes	Codes
1. Perceiving queer couples within the framework of gender binary paradigms	1.1 Appearance		Wears, behaves like, is like, appears, dressed as
	1.2 Live like husband and wife		Live like husband and wife, similar to/consider husband and wife, father, husband, wife, bride, groom
2. Queer people’s uninhibited and predatory sexual conduct	2.1 Queer people’s uninhibited sexual conduct	2.1.1 Onlookers’ accounts	Engrossed in each other, intimate moments, objectionable conditions, dirty activities, misdeeds, shame, deteriorating atmosphere, public display of affection
		2.1.2 Journalistic portrayals and interest in the sexual lives of queer individuals	Spent time together, had fun, growing closeness, sexual relationship, sexual desire, love affair
	2.2 Queer people’s sexually predatory behaviour		Physical overtures, inappropriate advances
3. Abhorrent and frenzied queer love			Illicit, unlawful, illegal, immoral, infamy
4. Queer relationships: an alleged cause of suicide			Due to/following/resulting from a homosexual/lesbian relationship
5. Family’s rejection of the deceased’s queer identity	5.1 Narrative of a close friendship		Friends, close friendship, just good friends
	5.2 Converted to queer narrative		Forced or brainwashed into becoming queer
	5.3 Straight ally narrative		Ally involved in activities of rehabilitation and improvement of the queer community
	5.4 The deceased was not a queer person		Parents’ outright rejection of the queer identities of their children
6. Deficiencies in suicide prevention interventions	6.1 Wrong information		Sweeping conclusions, faulty analogies, use of incorrect terms or descriptions
	6.2 The fault of the deceased		Victim blaming
7. Pessimistic and dreading tone			Deprived, depression, hate, searing indictment, rampant, dangerous, unsympathetic environment, everyday bullying, tragic pattern, fringes

### 4.4 Analysing themes

Through the lens of queer theory, the emerged themes, sub-themes, and sub-sub-themes were analysed. Use of this theory also helps shed light on the reason for the poor portrayal of queer people, which is due mainly to the fact that queer people defy the binary categories of gender and sexuality and the set norms of heteronormativity.

### 4.5 Drawing conclusion

In this step, the categorised news report statements were analysed to draw conclusions and provide explanations for the observed instances of poor reporting in queer suicide news reports.

## 5 Discussion

### 5.1 Perceiving queer couples within the framework of gender binary paradigms

The gender binary is regulated by the dominant force of heterosexuality, which sees the couple and relationship in a masculine and feminine binary (Butler, 2011). The same is reflected in a newspaper when the reports make queer couples intelligible to the dominant heterosexual readership and themselves by assigning male and female roles, relying on appearance, clothing, behaviour, and other stereotypical readings of queer individuals. In these reports, the media tends to suggest that queer couples resemble opposite-sex couples in behaviour and/or appearance, even when such details are irrelevant to the suicide case.

#### 5.1.1 Appearance

Examples of gender binaries created through clothing include:

1. “Mrs. Mehta wearing a male pullover and pants, and Mrs. Pant clad in a sari” (India Today, 2015).
2. “Bobby used to dress and behave like a man, whereas Puja was like any other girl” (Chakraborty, 2011).
3. “Sunita, a tomboy who preferred to wear shirt and trousers and sport short hair” (TNN, 2016).
4. One [woman]…used to wear pant-shirt^3^ (Dainik Bhaskar, 2018b).
5. It is worth noting that…Shobha…always dressed like men and behaved like them (Nai Dunia, 2016).
6. Diseased Gudiya Patro alias Raj Patro lived in the disguise of a boy… (Kumar, 2018).

The use of the term “disguise” in the last quote suggests that the clothing of queer women, which is perceived as masculine, is seen as an attempt to deceive or harm others.

In one report, the police share their investigative observations with journalists, considering it a possible clue to solving the suicide mystery: Station Officer Titawi has stated that Julie, a resident of Mawana’s Jhinjhar village, has been consistently wearing boys’ clothing from the outset. Similar to boys, she operates a taxi and also takes photographs dressed in men’s attire (Amar Ujala, 2020).

However, the reporting becomes even worse when journalists interpret the queer person’s deceased body, found after the suicide act, based on binary gender lines, as in the following cases:

1. “‘This morning, the bodies of Swapna and Sucheta were found in a field in the village. Sucheta was wearing a sari while Swapna was in trousers and a T-shirt,’ the officer added” (The Telegraph, 2011).
2. “Station House Officer, R B Tripathi said, ‘One of the girls was dressed in bridal wear while the other was clad like a groom’” (News18, 2005).
3. “Prasad dressed himself in bridal finery and wrote his lover’s name in red on his forehead before taking the extreme step” (The Indian Express, 2012).

The mention of clothing that is not aligned with the sex into which a queer person was born is so important to reporters that, in one instance, it appears in the headline: “Disappointed in love, homosexual dresses up in wedding finery, ends life” (The Indian Express, 2012).

Furthermore, another news portal dramatically narrates how the queer person dressed up before the suicide act:

The rest of the village was engrossed in a wedding taking place in a nearby hamlet, while Gudda, a young dancer in a Ramayan ‘mandli,’ secluded himself and began adorning attire resembling that of a bride. He chose a red saree accompanied by coordinating jewellery, applied a ‘bindi,’ lipstick, and makeup, and inscribed the name of his beloved on his forehead, before allegedly hanging himself in the cowshed of a neighbour (Gupta, 2012).

This report extends beyond discussing the external clothing of the queer body found at the scene of death and delves into the inner wear of the deceased person.

In another report, people wonder why a ‘good’ youth would die wearing women’s clothing:

The family members informed the police that Dipesh’s behaviour was respectable, and he had never been engaged in alcohol or any other form of intoxication. In addition to the family’s statements, the police are also questioning why Dipesh chose to commit suicide while wearing a saree and assuming a woman’s appearance (Haider, 2022).

This suggests that gender transgression is viewed negatively. It is baffling that the focus of the police and family members is on the queer youth’s clothing at the time of death instead of on addressing the intolerance that claims lives. Moreover, this report directly stigmatises queer people, portraying them as evil and immoral. Other reports hint at this by mentioning the individual’s appearance, which contradicts their assigned sex at birth.

In another report, besides clothing, the behaviour of a queer person that deviates from the behaviour of the sex to which the person was assigned at birth is described: One of them had an appearance and attire resembling that of a boy. Her gait and the cap on her head gave the impression of a boy. Interestingly, she displayed a deep concern for her friend, never letting her friend out of her sight (Bhatt, 2018). This description highlights who played the masculine role in the relationship, showcasing their protective nature towards their queer partner in case it was not evident from their clothing.

#### 5.1.2 Live like husband and wife

In another set of reports, the description of clothing and behaviour contradicting the sex to which one partner in a same-sex couple was assigned at birth is not sufficient to convey to readers the nature of the couple’s relations. Hence, these reports describe queer couples as “living as husband and wife” (PTI, 2006) or assign them husband and wife roles. For instance:

1. A gay couple named Raj Patro, alias Gudiya Patro, and Rashmita Nayak, who are living as husband and wife… (Kumar, 2018).
2. “It appears they were inseparable and loved each other like a man and a woman do” (Dasgupta, 2011).
3. Both used to communicate with each other in a manner similar to that of a husband and wife… The accused, Narayan, would address Vishnu as Billu and Babu regarded him as his husband (Dainik Bhaskar, 2018a).
4. “…a same-sex couple lived together as husband and wife for eight years…” (Tomar, 2020).
5. The deceased bodies of two young women, who had been living as husband and wife for a week, were found hanging together from a noose (Dainik Bhaskar, 2018b).
6. “Seeta went missing this afternoon and her 18-year-old ‘husband’, Vandana, has been locked inside her home” (The Telegraph, 2006).
7. “Twenty-one-year-old Baljit Kaur, the ‘husband’, took the step in the court of Judicial Magistrate Dimple Walia” (PTI, 2007).
8. “Pinky, the ‘father’, says she is confident of supporting the two-year-old on the money…” (Dave, 2008).
9. During the investigation, family members revealed that Premprakash Lodhi (23), a resident of Indira Nagar and a friend of Vishnu, had established a homosexual relationship with him. Both considered each other as husband and wife (Dainik Bhaskar, 2018a).

It is evident from these examples that even when the reports mention the queer couples’ relationships, they tend to use the familiar “husband and wife” terminology, reflecting a heteronormative perspective. Despite the fact that the English language offers gendered and gender-neutral options, reporters consistently prefer the husband and wife binary. This preference is also noticeable in a headline: “Kolkata model Bidisha De Majumder’s death mystery deepens as her ‘wife’ comes into the picture” (Chowdhury, 2022). The journalist could have used terms like “girlfriend” or “partner” but chose “wife” based on “specific evidence”:

[A] friend of the late model shared that Bidisha used to refer to the woman (seen in her Facebook photo) as “bou” (wife)….Bidisha also used to put sindoor on the woman’s forehead during their bridal photoshoots, claiming that only she could do that to her “wife” (Chowdhury, 2022).

Similar to the above quote, the use of wedding rituals to determine who was the bride and who was the groom in a queer couple is repeated here: “[T]he girl brought her 20-year-old ‘bride’ with ‘sindoor’ (vermillion) on her head to live in her Jawaharpuri house on Tuesday evening… The ‘groom’ was then locked inside a room and allegedly consumed some insecticide kept there” (PTI, 2006).

Additionally, one report incorrectly suggests that a couple’s behaviour and gender roles changed after they fell in love: Initially, the two shared a profound friendship, but their bond gradually transformed into love. In this relationship, one girl began to adopt a more masculine demeanour, while the other embraced a more feminine expression (Kadir, 2016).

Regardless of whether news reports focus on the appearance, behaviour, or gender roles of queer couples in an attempt to fit them into a heterosexual mould, it is evident that reporters are preoccupied with identifying a man and a woman in the relationship. Such practices reflect the entrenched nature of binary gender paradigms and the extent to which heteronormativity pervades society, even influencing news reports on queer individuals’ suicides. At a broader level, the danger of this practice lies in the fact that it perpetuates the restricted and oppressive nature of the gender binary while also erasing the lived experiences and identities of queer individuals (Casper et al., 2022).

In the context of queer suicide, such reports implicitly link deviations from traditional gender expressions to the deaths of queer individuals (Ghosh, 2022, p. 4), thereby shifting blame from societal norms to the individual’s gender expression. This phenomenon is reminiscent of how the 1980s U.S. news media portrayed gay men; victims of bullying were often depicted as violating societal gender-role expectations (Paceley and Flynn, 2012, p. 3).

Furthermore, the perpetuation of binary divisions reinforces the erroneous belief that “a queer person can be recognizable by certain attributes, which might be tastes, aesthetic appreciation, particular looks and glances, ways of moving or holding the body, ways of speaking, career choices or other behaviors” (Cover, 2012a, 12). This erases diversity within the queer community, leaving no room for non-normative identities and relationship patterns. To fit into this framework, individuals must adhere to the hierarchical gender structure, perpetuating stereotypes by reducing queer couplehood to one-dimensional portrayals, with one person being feminine and the other masculine, despite the fact that this might not reflect reality.

Queer theory criticises this practice and emphasises the multiplicity as well as the flexibility of gender and sexuality beyond the realm of gender binaries. It is also not in agreement with the notion that relationships should be based on hierarchy or any such idea. Instead, it highlights individuals’ power to determine their own identities and characteristics of relations (Regan and Meyer, 2021). However, this independence is removed when assigning gender identities according to heteronormative norms and binary paradigms.

A news organisation should never use media platforms to reinforce heteronormativity via a binary division which would be unacceptable conduct from a responsible media house. Rather, media should focus on providing an accurate representation of queer relationships without reinforcing negative stereotypes. This includes news articles that are characterised by inclusivity, intersectionality, and recognition of differences among experiences within the queer community. This will entail challenging harmful stereotypes as well as breaking down gender and sexual orientation stereotypes; privileging voices from marginalised groups; and representations that encompass all forms of human expression and identity.

### 5.2 Queer people’s uninhibited and predatory sexual conduct

The media’s hyper-sexualisation of queer people, particularly women, is not a new phenomenon (Randazzo et al., 2015). From the early days of the AIDS epidemic, when the disease was called “Gay-related Immunodeficiency” (Altman, 1982), to the more recent ban on blood donations from queer people (Jensen, 2021), there has been a tendency to portray queer people as engaging in more promiscuous and reckless sexual behaviour compared to their straight counterparts.

The news reports also contribute to this theme, focusing on queer people’s sexual lives and intertwining their stories of suicide with their intimate relationships. Journalists seem particularly interested in depicting queer people’s (1) uninhibited sexual conduct and (2) sexually predatory behaviour. Both aspects are discussed in detail below.

#### 5.2.1 Queer people’s uninhibited sexual conduct

Reports often use two approaches to describe queer people’s careless sexual activities. First, they quote onlookers who describe how the queer individuals were caught or appeared to be displaying uncontrollable sexual behaviour. Second, the journalist becomes an omnipresent narrator describing a queer couple’s sexual conduct in the absence of onlookers. Journalists use both approaches to describe intimate details and indirectly indicate that the queer people deserved the fate of death because they had lost sight of the right path and were walking in the wrong direction. Each approach is described in detail as follows.

##### 5.2.1.1 Onlookers’ accounts

This approach includes eyewitness accounts of queer people’s sexual acts to describe how they were caught engaging in sexual behaviour or public displays of affection, hinting at their careless and unconcerned behaviour. The witnesses or onlookers can include various individuals connected to the deceased queer person, such as friends, parents, neighbours, colleagues, classmates, relatives, and acquaintances. The following quotes illustrate onlookers’ descriptions of queer couples indulging in homosexual conduct:

1. “‘…they were so engrossed in each other that they used to draw attention and vulgar connotations… The girls kept meeting and having their lunch and dinner together from the same plate,’ said a relative” (Chakraborty, 2011).
2. One day on Mumbai’s Marine Drive, a relative of Rujukta saw them both engrossed in intimate moments (Saxena, 2018a).
3. “After falling in love the prisoners duo developed the relationship and many a times were found in objectionable condition[s] in jail” (FPJ Bureau, 2019).
4. “‘We have been told that they had started locking themselves in the bathroom for hours…,’ said PI [Police Inspector] M A Singh” (Hindustan Times, 2018).
5. Your daughter was involved in dirty activities (Kumar, 2019).
6. Pahade stated that investigations have unveiled that one of the girl’s parents recently discovered the duo in a compromising position (Tiwari, 2008).
7. The family informed us that they had previously witnessed both of them in an objectionable position (Dainik Bhaskar, 2018a).
8. Some time ago, both were found in an objectionable condition (Sharma, 2021).

The last quote is taken from the drophead (deck) of the report, possibly to encourage readers to delve further into the body of the report or because of the journalist’s misplaced belief that the matter was important enough to deserve a place in the drophead.

The reports also shed light on the negative impact of the queer individuals’ behaviour, which ranges from causing discomfort to others to adversely affecting the entire village. For instance:

1. “‘They were so intimate that we did not feel comfortable chatting with them,’ said a classmate of Puja” (Chakraborty, 2011).
2. The villagers reported that both of them had held the family’s shame in abeyance. Due to the couple’s misdeeds, the atmosphere of the village had also started deteriorating (Raghuwanshi, 2023).

Queer couples are either punished or warned of the consequences if they repeat such behaviour. For example:

1. “We have been told that they had started locking themselves in the bathroom for hours. Fearing that their behaviour would adversely affect other employees, their employer sacked the duo,” said PI M A Sing (Chauhan, 2018).
2. “…said Gautam, admitting that they would also reprimand the couple for a public display of affection” (Chakraborty, 2011).
3. “The girls were reprimanded and asked to maintain distance” (Tiwari, 2008).
4. “Swapna and Sucheta had been cautioned by villagers on several occasions” (The Telegraph, 2011).

These quotes suggest that the victims—and not the perpetrators of the violence—are being punished. Moreover, the dominant group justifies the punishment inflicted upon them by attributing it to their alleged bad behaviour.

##### 5.2.1.2 Journalistic portrayals and interest in the sexual lives of queer individuals

It becomes increasingly evident that journalists are fixated on queer people’s sexual lives when they include information, of questionable veracity, that portrays them In a negative light, even In The absence of eyewitness accounts confirming such behaviour:

1. “The two also had a physically intimate relationship for a long time” (Mirror Now Digital, 2022).
2. They both enjoyed each other’s company, and when they openly discussed their sexual desires, a love affair was formed (Saxena, 2018b).

In some cases, the intimate details are so important that they appear in the headline. To quote the first instance: Two lesbian daughters-in-law fell in love…engaged in physical relations, both died within 15 min (Raghuwanshi, 2023). Here, the use of the familial relationship term “daughters-in-law” in the headline intensifies the perceived severity of their supposed crime, suggesting that they crossed the societal boundaries that a married woman with a family is not supposed to cross. Similarly, in the second instance, the following headline hints at the queer couple’s supposed immorality and evokes sensationalism: Young man co-habits with eunuch in a room for 6 years and takes drastic step afterwards (News Desk, 2019). However, the body of the report clarifies that the youth was in a live-in relationship with the transgender person for 6 years (News Desk, 2019). The headline implies a different meaning to evoke curiosity.

One report links intimate moments to suicides or tragic events: One day, Bhavna’s husband caught sight of her sharing an intimate moment with her friend. The distress from this sight drove him to commit suicide due to overwhelming shame (News18, 2018). However, another news report on the same incident attributes his suicide to the fact that his wife ran away from the house: Kanti was deeply troubled by his wife’s attraction to another woman. Tragically, he took his own life when his wife left their home on June 8th (Chauhan, 2018). The comparison of different news reports on the same incident reveals a tendency to sensationalise the suicide story at the expense of queer people.

Journalists do not always approach the narration of queer people’s intimate lives in a direct manner. Sometimes, sexual relationships are not explicitly stated but, rather, are hinted at:

1. The two girls spent a substantial amount of time together (Kadir, 2016).
2. Their friendship bore fruit, as Rujukta and Roshni spent entire days together, having a great deal of fun. Before long, a romantic relationship blossomed between the two (Saxena, 2018a).
3. It’s likely that he used it to open the shop every Sunday night after business hours, a place where the two friends were frequently seen spending time (Kannan, 2018).

In one case, the journalist attempts to estimate the frequency of sexual intercourse between a queer couple: The two also had a relationship several times. Since Nikki used to be in relationships with other boys as well… (Rawal, 2022).

Furthermore, in one report, homosexuality is depicted as a drug to which people fall prey, leading them to act wildly and lose their senses: The case involves two female students who were deeply involved in homosexuality to the extent that, despite opposition from their family members, neither could restrain their feelings (One India, 2011).

One report suggests that the police want to investigate and conduct medical tests to find out if the couple had engaged in sexual relations: “Police ha[ve] sent the two bodies for *post mortem* and also pla[n] to conduct medical tests to find out if they had physical relations” (TNN, 2009).

In cases of rape and sexual assault, medical tests are necessary, but in cases involving consensual relations, such tests seem illogical and raise concerns about the media and law enforcement’s peculiar interest in the sex lives of queer people. In the broader context, portraying the couple’s intimate behaviour as uncontrollable and providing subsequent warnings and punishments are means of absolving the instigator for their actions.

Lastly, news stories that depict the queer population as having uncontrollable and predatory sexual behaviour only serve to validate existing stereotypes and biases against queer individuals, ultimately perpetuating the stigmatisation and marginalisation of this community. Queer theory refutes the claim that any non-heterosexual behaviour is, by definition, abnormal. When the news speaks about the lack of restraint in the sexual activities of queers, they are also solidifying negative assumptions that queer individuals are essentially deviant, aggressive, or ill.

Therefore, representing queer individuals having sex freely promotes a sexuality-focused perspective according to which queer individuals are sexual objects and not human beings with lives beyond their bedrooms. Additionally, it adds to the misbelief that queer sexuality is all about sexual desire without considering the emotional, social and cultural realms of queer people.

Moreover, sensationalised media reports that make unrestrained sexual behaviour among queer people part of a headline may be seen as supporting prosecution or persecution among those populations. Such sensational propaganda supports the introduction of discriminatory practices and policies aimed at further strengthening the suppression of civil liberties.

#### 5.2.2 Queer people’s sexually predatory behaviour

Predatory sexual behaviour is considered the most severe type of crime a person can commit, and strict laws are in place to address such criminal conduct. However, some reports attempt to equate queer people with this crime by accusing them of displaying predatory behaviour. In such narratives, friends, roommates, and authorities of deceased queer individuals make accusations of sexual advances. For instance:

1. “Kamran had made several physical advances by touching his body, kissing and hugging” (Firstpost, 2013).
2. “The police said university authorities had [said] they had received complaints against Kamran from many other students” (The Indian Express, 2013a).
3. “At least two junior students had accused Ajay of making inappropriate advances” (Ghatwai, 2012).

Such angles perpetuate the belief that queer individuals are sexually animalistic deviants who cannot control their urges and who engage in predatory behaviour that endangers those around them. This narrative implies that queer individuals bring death upon themselves by crossing the boundaries of appropriate sexual conduct. Queer theory critiques these essentialist perspectives on sexuality and gender by emphasising the variety and complexity of human experiences and challenging the heteronormative belief that queer individuals are inherently deviant or predatory due to their sexual orientation or gender identity.

Furthermore, the practice of depicting queer individuals as sexual predators in the context of suicide overlooks the structural concerns—such as systemic prejudice, violence, and marginalisation—that contribute to mental health problems in queer populations (Moagi et al., 2021). A study on cyberbullying indicates that victims are often blamed for the bullying they endure, particularly when their sexuality is viewed as deviant (Thornberg, 2015). Similarly, news reports on suicide often blame deceased queer individuals for their own deaths, portraying them as shameful and immoral citizens rather than respectable ones. This flawed logic shifts responsibility away from those who drive queer individuals to suicide and onto the individuals themselves, “cast(ing) suicidal subjects as ‘Other’” (Roen et al., 2008, p. 2089). By framing queerness as implicitly causing suicide, these stories become more about deviancy than suicide (Cover, 2012b, p. 1,173). Furthermore, the portrayal of queer people who commit suicide as sexual predators contributes to the criminalisation and persecution of the queer community, perpetuating the idea that queer people are a danger to society.

Moreover, when these derogatory accounts appear in news reports, which are considered nonfiction and factual, there is a higher likelihood that people, especially queer young individuals who often rely on newspapers as their primary source of information on suicide, will believe them to be true (Cover, 2012b, p. 1,173).

To sum up, the news stories about queer suicide are presented in a way that exposes their bigotry and oppressive views by showcasing the uncontrollable and immoral sexual behaviour of individuals. It highlights the importance of challenging heteronormative assumptions and advocating for more authentic and inclusive media portrayals of queer lives, struggles, and successes.

### 5.3 Abhorrent and frenzied queer love

Sedgwick (2008) mentions age-old arguments against homosexuality, comparing it to paedophilia and bestiality, in order to reject and pathologise it. This demonisation is reflected in news reporting that portrays queer individuals as blinded by immoral love. For instance:

1. The police assert the existence of an illicit relationship between the two (Mirror Now Digital, 2022).
2. Hari Om’s family has also acknowledged the relationship and the illicit nature of their connection (Yash Bharat, 2022).
3. Indeed, an unlawful physical relationship existed between the two (Zee News, 2022).
4. The entire incident unfolded in an attempt to prevent the exposure of the secret illegal gay relationship, which would tarnish their reputation in the area (Rawal, 2022).

In these reports, the use of terms like “illicit,” “illegal,” “homosexual,” and “physical” highlights the criminal and/or sexual nature of the relationships when a simple mention of the relationships would have sufficed. It is worth noting that although the Indian High Court decriminalised same-sex relations in 2018, newspapers continue to criminalise them.

In one report, the journalist expresses disbelief that same-sex individuals can form emotional bonds with each other, using the word “confess” to describe the father’s acknowledgement of his daughter’s emotional relationship with another girl: Her father also confessed that his daughter had an emotional relationship with the deceased girl. We are curious about how two girls can forge such an emotional bond between themselves (Janta Se Rishta, 2022).

Another report begins with an irrelevant introduction, setting the stage for a tragic queer love story by depicting the irresponsible and rebellious love of the current generation:

In recent times, numerous incidents are frequently linked to boyfriend-girlfriend, wherein young couples meet, interact, and eventually fall in love. These lovers become so deeply engrossed in their love that it becomes their primary focus, often leading to conflicts with their families. Alarmingly, even young children are getting entangled in relationships that disrupt their lives. Alongside these common narratives, there are also unusual love stories that surface, resembling extraordinary tales. Currently, there’s a discussion surrounding the tragic suicides of a transgender individual and a married young man (Naman, 2022).

Another report illustrates the heartlessness of a queer mother who sought shelter with her partner while her daughter cried from hunger: One day, they both contemplated escaping and departed for Ahmedabad. During their stay there for two nights, they struggled to find shelter. Asha’s young daughter was also crying due to hunger (News18, 2018).

In one instance, a report blames a queer person’s love for the death of a family member, implying that the relationship caused her passing: “It needs mention here that Ankur’s mother had passed away 15 days ago and family members cited the duo’s relationship as the reason for her passing away” (The Sentinel Assam, 2020).

Certain headlines sensationalise queer relationships, linking them to murder and suicide:

1. What kind of relationship is this? What kind of bond is this? A shocking true incident of brutality and murder in an immoral relationship with a transgender person, followed by suicide (The Sastra, 2022).
2. Murder in sexual relationship with transgender person: Accused hangs himself in fear of infamy (Zee News, 2022).

In some cases, journalists justify parents’ attempts to ‘cure’ their queer children, even if it leads to suicide. For example: Queer girl’s uncle justifies the extreme measures, stating that the family wanted to give her a “good life”: “[U]ncle, Ajit Mondal, says, ‘Let the police do what they want. We wanted to give her (Sucheta) a good life. She chose to die’” (Dasgupta, 2011). Another news report mentions that the uncle’s intended “good life” for her involved marrying her off and separating her from her partner (The Telegraph, 2011).

Thus, another frame emerging from queer suicide reporting is the portrayal of queer love as wrong, followed by attempts to persuade individuals to conform to societal norms. This theme can be attributed to the belief that presenting compelling social narratives based on cultural mores would increase audience interest in suicide (Armstrong et al., 2020, p. 14). Consequently, suicide stories in these reports often incorporate elements of queer love and immorality, which defy traditional norms, with the aim of generating and increasing readership. Hence, news stories depicting queer individuals who commit suicide as engaging in abhorrent and frenzied love reveal the underlying prejudices and preconceptions that such narratives perpetuate.

Queer theory counters the common belief that queer love is abnormal simply because it is divergent from heterosexual standards. By depicting it as abhorrent or desperate, media coverage only serves to reinforce stereotypes and perpetuate the notion that all queer relationships are inherently unhealthy or unsafe. Meanwhile, this narrow focus on individual actions undermines efforts to address the structural inequalities that contribute to disparities in mental health among queer populations.

Furthermore, the representation of queer individuals who take their own lives due to deplorable or crazed love leads directly to the path of criminalisation and persecution of the queer population. It sustains the narrative that queer love is a threat to society’s standards. Therefore, it reinforces acts of discrimination and legislations that worsen the conditions of inequality and victimisation experienced by queers in society. By addressing structural injustices and fostering empathy as well as acceptance, news reports can contribute to developing an environment supportive of queers dealing with mental disorders. This framing is closely linked with blaming queer relationships for suicides, which will be discussed next.

### 5.4 Queer relationships: an alleged cause of suicide^4^

That queer people are blamed for their suicides is a well-documented reality (Hein and Scharer, 2013; Finnigan, 2015). Similarly, queer suicide news reports often portray queer relationships as the primary cause of suicide, suggesting that a queer person’s identity—and not society’s discriminatory attitude towards queer individuals—leads them to take their own lives. The headlines of these reports, which essentially say, “Life lost due to homosexual relationship,” suggest that homosexuality is to blame. In contrast, the body of the reports might reveal a different underlying cause. For instance:

A young man took his own life following a homosexual relationship. [Headline] The family revealed that the accused young man had issued threats to his partner, stating that he would either end his own life or harm his partner if the latter decided to marry. Overwhelmed by fear, the partner eventually took his own life [Body] (Dainik Bhaskar, 2018a).

Here, the report’s headline blames a young man’s suicide on his homosexual relationships, while the body of the report mentions that his actions stemmed from threats from his lover.^5^ In another report, the headline links a man’s suicide to his depressed state over a homosexual relationship, while the body reveals that blackmail was the true cause:

Depressed over homosexual relationship, man commits suicide [Headline]…The suicide note discloses that the young man was involved in a homosexual relationship with [a] 15-year-old minor boy, who allegedly engaged in consistent blackmail. As a consequence, the young man tragically took the life of the minor boy before ending his own [Body] (Chhattisgarh Darpan, 2022).

Furthermore, in one report, the headline implies that queer relationships lead to HIV infection, which then causes suicide. However, in the body of the report, it becomes evident that a threatening message from the person’s lover was the reason for the suicide:

Surat: Saree-dress material businessman dies by suicide amid mental stress from HIV resulting from homosexual relationships [Headline]…The Varachha police have filed a case of abetment to suicide following a threatening message from Amandeep, who was involved in a homosexual relationship with the deceased individual, as well as the presence of a suicide note left by the young man [Body] (Loktej, 2022).

In the subsequent quote from the report, the opening paragraph attributes the suicide to the relationship: A shocking incident has occurred in Mumbai, the country’s financial capital, that has left everyone stunned. A case of suicide by a girl due to [a] homosexual relationship has come to light (Kadir, 2016).

Similarly, another news report includes a quote from a police official who links the suicide to the relationship: “‘The two women committed suicide due to complications arising out of their lesbian relationship,’ the official said” (PTI, 2018).

This quote can be interpreted as suggesting that a non-heteronormative relationship leads to complications because it deviates from societal norms. Such an explanation does not address the real issues, such as homophobia or heteronormativity. The resulting implication is that not only journalists but also some police officials lack awareness and understanding.^6^

The same framing continues in the following quote: “The individual who murdered the young man also took his own life by hanging. The motive for the murder was reportedly attributed to the immoral relationship between the two” (Vishwakarma, 2022).

Another news report reinforces the notion that such relationships are immoral by describing how a relationship led to the downfall and death of an individual in the past:

A similar incident had emerged a few years ago. In Betul, a young man inflicted serious injuries upon himself due to his affection for a transgender individual. Indeed, an illicit relationship existed between the young man and the eunuch. In the course of their relationship, a dispute arose between them, leading the young man to attempt suicide in the presence of the eunuch (Zee News, 2022).

Newspapers’ shallow understanding of the cause of suicide is also evident in this case:

The investigation revealed that the deceased, HariOm Chaure, had been involved in a homosexual relationship with a transgender person named Nikki. The entire incident unfolded only in an attempt to prevent the exposure of the illicit homosexual relationship, which would tarnish his reputation within the community (Rawal, 2022).

However, the report fails to examine why such a relationship is considered illegal despite the decriminalisation of section 377 or why its disclosure would bring shame to queer couples. It overlooks important questions about the underlying heterosexism, which is the real issue, not homosexuality.

In another incident, the news report initially mentions the reason for suicide as being a homosexual relationship. However, upon further reading, one discovers that the real cause was the depression resulting from the individual’s ‘friend’ ceasing communication with him:

In the suicide note, the student expressed that he was distressed due to a homosexual relationship and subsequently hanged himself… Over the course of a few months, Naman’s friend ceased communication with him, leading him into a state of depression (Singh, 2022).

A report covering a separate suicide pact blames the homosexual relationship for the breakdown of a marriage: As a result of her homosexual relationship, a woman began residing at her maternal home instead of her in-laws’, eventually leading to the breakdown of her marriage (Raghuwanshi, 2023). The report fails to address such crucial aspects as whether the marriage was forced, the woman’s happiness in the marriage, and whether the husband and wife loved each other and were compatible. By attributing the breakup to homosexuality, i.e., linking one negative aspect to another, the report perpetuates the stigma surrounding both homosexuality and divorce.

Another inappropriate headline is as follows: The two women fell in love while working together in the office, and their bodies were discovered 1 day (News18, 2018).

While the headline itself does not directly blame queer relationships for the suicides, the reporter mentions two events: the development of a romantic relationship and the tragic discovery of the deceased lesbian couple. This connection can lead readers to falsely assume that one event caused the other. Instead of shedding light on the discrimination the individuals might have faced due to their identity, the report body sensationalises the matter by mentioning unverified information, such as implying that the couple was caught in a compromising position or portraying the mother as callous and insensitive for leaving her daughter hungry (News18, 2018). These negative portrayals contribute to the objectification and hatred directed towards the queer mother.

News headlines blaming queer relationships for suicide reveal underlying biases and heteronormative assumptions. This finding, suggesting a link between queer relationships and suicide, is consistent with other research indicating that the perceived deviancy of non-heterosexuality may contribute to suicidal tendencies (Cover, 2012b, p. 1,173). Queer theory contests this idea that queer relationships are inherently dysfunctional due to their divergence from heterosexual norms. The media reports on suicide associated with queer unions reinforce prejudiced thinking, which further supports the falsehood that queer individuals cannot have strong and meaningful relationships.

Moreover, assigning suicide to queer partnerships worsens the opprobrium and seclusion of queers. It sustains a notion that queer relationships are naturally weaker or maladjustive, thus being supportive of the prejudiced outlooks and practices leading to more marginalisation of queer people.

Because of the above implications, journalists should understand that connecting sexual perversion with suicide cases of queer individuals is inappropriate. Reporters should avoid attaching suicide to unreal or simplistic causes, as it does not solve the underlying problems. Instead, they need to emphasise the links between heteronormativity and its structural violence towards non-heteronormative individuals and relationships. If people do not talk about heteronormativity and how it adversely affects the queer community, violence will continue unabated (Greene, 2013, p. 14).

### 5.5 Family’s rejection of the deceased’s queer identity

The following quote, extracted from a news report, describes a deceased transgender individual; one segment of it inspired the title of this paper:

“The hostel authorities told Mirror that Fathima liked to dress like boys. She also had her hair trimmed. She was a ‘nalla kutti’ (good child) otherwise, the source said” (Emmanuel, 2018).

This quote implies that society perceives queerness as an undesirable trait. Consequently, it is unsurprising that families often seek ways to reject it. Past studies have focused primarily on family acceptance or rejection of queer individuals after they come out (Baiocco et al., 2015, 2016). However, the news articles in this study shed light on a different aspect: the deaths of children and how those deaths have drawn attention to their sexuality, which some parents might have been aware of or discovered only after the tragic event of suicide. These news reports either completely ignore the queer aspect of the individual’s sexuality, hint at it subtly in the subtext, or ambiguously portray it.

Alternatively, the newspapers do not comment on it but quote both sides of the argument, for instance, families’ insistence that their children are not queer, contrary to activists’ comments or the deceased’s pre-death revelation of their non-normative sexuality. This parental refusal stems from the shame that the family feels if the deceased is identified as queer.

To justify this denial, news reports indicate one of the following three reasons: (1) the deceased was close friends with another queer person who either was deceased or survived; (2) the deceased was allegedly forced into queerness by another queer person; (3) the deceased was a straight ally of the queer community; and (4) the deceased was not a queer person. These reasons are discussed further below, with relevant quotes taken from reports.

#### 5.5.1 Narrative of a close friendship

When it comes to a queer couple in which both partners or one partner have died by suicide, news reports refer to them as just friends, while other details in the reports hint at their actual relationship. Following are quotes from suicide reports that downplay the couple’s relationship:

1. “Two women committed suicide by hanging themselves… Both were friends” (TNN, 2020).
2. Two girls who shared a close friendship allegedly took their own lives by hanging themselves simultaneously in their respective rooms using scarves (Baluni, 2019).
3. In the Muzaffarnagar district of Uttar Pradesh, two friends passed away after ingesting poison (Kapil, 2020).
4. “Under what looked like a suicide pact, two lady teachers…jumped off a drop of 95 ft.… What had apparently upset the two year old friendship of the two women was the transfer order…” (India Today, 2015).

Previous studies have confirmed that India has witnessed a number of double suicides or suicide pacts by female lovers who seek to avoid separation from their partners and subsequent forced marriage to a man (Vanita, 2005; Ghosh, 2022). While some reports provide no information other than describing them as friends, others subtly hint at the individuals’ relationship. For instance, in one report, the only clue to the couple’s relationship is their death by a suicide pact and the fact that they were close friends (TNN, 2020). In another report, several phrases suggest their relationship, including the reason for their suicide being the transfer order of one of the colleagues, phrases like “till death do us part,” the use of “companion” instead of “friends” in certain sections, and statements like they “used to spend the evenings together while their husbands were away on work,” along with their photos published with the report (India Today, 2015). In one report, the parents of one of the girls vehemently denied “homosexual theory” and maintained that their daughters were “just good friends” (Kumar, 2019).

Furthermore, in three news reports about a suicide involving a queer youth, one report refers to the person he contacted before his death as a friend, while the other two reports refer to him as a boyfriend:

1. “Abhishek Lahane, Avi’s boyfriend… says, ‘On July 2, he called me around 5 pm and said he is going to kill himself, without giving any reasons’” (Gowalla et al., 2019).
2. “Police said Avinshu had contacted one of his friends in Mumbai around 5 pm the same day and told him that he was going to kill himself” (TNN, 2019a).
3. “[A] police source said he had spoken to one of his friends in Mumbai the previous day and told the latter that he would take his life” (The Hindu, 2019).

Another news report attributes the suicide of a queer individual to his separation from the person he was in love with, who was getting married. The news report refers to them as friends, but it also indirectly hints at their relationship:

They told police that six months ago, Raval had gotten engaged. He was disturbed and used to tell the family that his friendship would end when he got married. The two began to stay together for longer periods of time (TNN, 2009).

It is possible that in some cases, the term “friendship” was used as a euphemism for the word “homosexual,” which is considered disrespectful.

Another news story, about a suicide pact between a transgender person and a man, consistently refers to their relationship as a “friendship” except in the concluding part:

In friendship, a eunuch and a young man took their lives by jumping in front of a train. [Headline] The police investigation uncovered that the young man had a friendship with the eunuch… Simultaneously, the police state that the young man was acquainted with the eunuch. They had been living together since September 12. Both of them likely took this step due to someone interfering in their friendship. [Body] It is believed that external factors may have led to this tragic decision stemming from their friendship [Ending] (Rohtak Bureau, 2019).

The following report mentions the friendship between two individuals in its headline, similar to the previously quoted report, and indicates that the end of this friendship was the cause of the person’s suicide. However, the body of the report implies that their relationship was more than a friendship: “Dumped by friend, youth ends life [Headline] Family members said that…[Himanshu] befriended a youth 2 years ago. A week ago, Himanshu’s friend refused to meet him under family pressure. Himanshu has been disturbed ever since” [Body] (Free Press Journal, 2022).

In another report, the police and the institution where the individual was studying claim that he was queer, though some students deny it:

Three days later, the police revealed that Kamran was in ‘love’ with his roommate and had even made several physical advances… Banners and posters contradicting the police claims… have come up on the campus. “Kamran was not homosexual,” says one such poster (Zee News, 2013).

In one news report, an activist opines, “Earlier, people would call these incidents ‘death of close friends.’ Now, the inhibition of using phrases like ‘lesbian lovers’ has gone” (Dasgupta, 2018b). However, despite this change in perception, several reports (Baluni, 2019; Gowalla et al., 2019; The Hindu, 2019; TNN, 2019a; Rohtak Bureau, 2019; Kapil, 2020; Free Press Journal, 2022) published after 2018 repeat the narrative of describing these individuals as merely good friends. This suggests that the practice of downplaying queer relationships by referring to them as friendships persists.

#### 5.5.2 Converted to queer narrative

Beyond the close friend narrative, another prevalent way to deny an individual’s queer identity is by suggesting that they were forced to become queer or were brainwashed into doing so. In one case, family members blamed the partner for the individual’s homosexuality, claiming that “his partner… had forced him to alter his lifestyle” (The Indian Express, 2011). However, it is important to note that another news report states that the person publicly came out as homosexual on a reality show (The Indian Express, 2011). Moreover, the headline of the same news report, “Man ends life, family claims he was forced to become gay,” (The Indian Express, 2011) emphasises the idea of forceful conversion, making it a crucial aspect of the event, according to the journalist.

In another report, the parents of the deceased assert that other transgender individuals influenced their child to undergo gender affirmation surgery and marry a man:

Raju did not exhibit typical feminine qualities since childhood. Recently, he connected with Aman Singh and transgender individuals. Two months ago, Raju underwent plastic surgery to undergo a sex change and subsequently married Aman Singh of Birsanagar (Dainik Bhaskar, 2018c).

These instances indicate a lack of understanding among queer individuals’ family members, who believe that someone can be converted to being queer. Such narratives might spread misinformation, suggesting that queerness is a contagious disease or that a person can be brainwashed or influenced into becoming queer. The media must provide additional information to readers, clarifying that external influences cannot make straight people queer, just as conversion therapy is ineffective in changing a queer person’s sexual orientation.

#### 5.5.3 Straight ally narrative

One news report portrays a queer individual as an ally, thereby masking their actual queer identity. The report highlights the fact that the person was involved in activities aimed at the rehabilitation and improvement of eunuchs and orphans. It reiterates this aspect twice. The report also includes people’s responses to the individual’s “social service,” using terms like “taunt” and “snide remarks” (TNN, 2013). Another report focuses solely on the queer person’s “interaction” with the queer community, which disheartened their family, while omitting any mention of their charitable endeavours (The Indian Express, 2013b).

The full scenario becomes apparent through the information contained in another news report, which cites an NGO worker familiar with the individual. This worker stated, “He is a transgender, and danced openly at the rally. TV channels broadcast the footage in the evening news. He returned home and a fight erupted” (Express News Service, 2013).

The newspapers’ portrayal of a queer individual as an ally presents an inadequate and skewed view. Newspaper journalists must engage with all relevant parties to ensure an accurate and comprehensive account of the story.

#### 5.5.4 The deceased was not a queer person

In the preceding three sections, the reports presented various explanations for the non-queer status of the deceased individuals, such as being friends with a queer person, being coerced into assuming a queer identity, or acting as an ally. This section reveals instances in which parents outright rejected the queer identities of their children. A suicide report concerning a queer couple illustrates the dichotomy between one girl’s parent accepting her emotional connection with another girl and the other girl’s parents adamantly denying it (Janta Se Rishta, 2022). In a different suicide case, one report includes a father’s assertion that his son was not gay but, rather, a “simple and religious person” (Namboodiri, 2019). To bolster this stance, the father, in another new report, indicates the pursuit of a heterosexual matrimonial match for his son (Ozarkar, 2019).

The notion that queerness contradicts morality and religious principles and that individuals of strong moral and religious standing would not choose such a path—as reinforced by marriage plans—forms the basis of the father’s argument. However, this reasoning is contradicted by the suicide note, which cites “taunting and teasing over his sexuality” as the reason for the individual’s suicide, thus challenging the father’s assertion that his son was heterosexual (Ozarkar, 2019). In two other news reports concerning a suicide pact, the families dismiss their daughters’ queer identities without presenting any counterarguments (News18, 2005; Dwivedi, 2019).

Overall, a family’s refusal to acknowledge the deceased’s queer identity underscores the challenges and prejudices that queer individuals and their relationships face in terms of acceptance, both before and after death, from their families and society. Activists emphasise that the stigma associated with queerness significantly contributes to suicides (TNN, 2016; Dasgupta, 2018a). Paradoxically, this shame sometimes persists even after a suicide, intensifying due to heightened media and public scrutiny of the act. Consequently, some family members deny this reality rather than confront it.

In addition, when a queer individual dies, the refusal of the family to accept their queerness proves that communities and families are adopting heteronormative ideas. Again, this indicates that people find themselves pressured to live up to society’s expectations regarding sexuality and gender. The absence of queer identities from both home and social environments fosters feelings of shame, isolation, and alienation in queer people (Garcia et al., 2020).

Journalists play a pivotal role in concealing the queer identities of individuals who die by suicide. They do this not merely to avoid offending readers but primarily due to the reluctance of the deceased’s family to reveal the deceased’s identity. Journalists may collaborate in suppressing the queer aspect of suicide reports or support the family in promoting a narrative that disregards the individual’s queerness. This approach deprives journalists of a critical opportunity to educate their readers and potentially prevent additional suicides.

### 5.6 Deficiencies in suicide prevention interventions

According to World Health Organization (2017) guidelines, news reports about suicide should include warning signs and preventive strategies to help those at risk. The inclusion of such information can benefit readers who require support, are dealing with suicidal tendencies themselves, or know someone who is. Unfortunately, queer suicide news stories often lack this essential information. On rare occasions, reports feature opinions and quotes from counsellors, psychologists, sociologists, activists, and individuals from the queer community who have overcome mental health challenges.

Conversely, at times, reports provided inaccurate information and misleading guidance and suggestions that could have been counterproductive and potentially worsened the situation. Examples of these can be broadly divided into two categories: (1) Wrong information: instances of disseminating incorrect information and (2) Fault of the deceased: cases in which the deceased’s actions are incorrectly attributed as the cause of the suicide.

#### 5.6.1 Wrong information

In this case, reports cite experts who provide incorrect information by making sweeping conclusions, faulty analogies, or using incorrect terms or descriptions due to their limited knowledge or lack of expertise in queer issues.

In one news report about a suicide pact, a psychologist attempted to convey the notion that queer love is equivalent to heterosexual love by stating, “Their emotions for each other would be the same as one would find in a committed heterosexual relationship” (TNN, 2006). This statement, which aims to promote acceptance of queer people, implies that queer relationships should be accepted only if they match the standards set by heterosexual couples. However, this raises the question of whether, to deserve acceptance, a bond between or among queer people must be similar (committed and monogamous, procreational) to the bonds in heterosexual relationships.

Another report quoted a psychiatrist and sociologist who reduced queer individuals’ freedom to love by describing it as “their sexual choices” (Dasgupta, 2018b) or “their preference” (TNN, 2006), respectively. Reducing a person’s sexual orientation to a choice or preference is misleading. Research has shown that sexual orientation is influenced by a combination of environmental, emotional, hormonal, and biological factors (WebMD Editorial Contributors, 2022) and cannot be attributed merely to a person’s choice.

In another instance, a doctor made the mistake of generalising information about the entire population of India based on a few interactions with queer people and consequently stating that “attempting suicide is a way of letting their parents know about their sexual orientation” (Express News Service, 2015).

Another news report cited an advocate who falsely claimed that “Indian law does not allow for same-sex marriage” (The Telegraph, 2006). In fact, Indian law does not recognise gay marriage but does not criminalise it, and many same-sex relationships openly exist in India (Jain, 2023).

In yet another report, an activist commented, “Earlier lesbians usually came from the elite class. But now, there is no class barrier as such” (TNN, 2006). A news report about a transgender woman who committed suicide quoted another transgender woman who said, “[E]very transgender wishes for sex reassignment surgery” (Antony, 2022). This statement is untrue and misleading, and it perpetuates a myth that must be debunked. The gender affirmation process varies from individual to individual and includes surgical and non-surgical procedures; not everyone requires surgery (Pallarito, 2023).

In conclusion, the inclusion of such comments in news reports is concerning. It highlights the lack of knowledge about the issues being reported. These misconceptions are spread widely, and readers might perceive them as factually correct. Thus, journalists should fact-check such statements before publication.

#### 5.6.2 The fault of the deceased

This category includes opinions and advice from activists and popular queer figures that often fall short of their intended purpose and are dishearteningly marked by victim-blaming. For example, a prominent queer figure offers advice under a sub-heading titled “Suicide is never the solution,” conveying the following message: “Our society still views deviation from the gender binary as a curse. Thus, it becomes our responsibility to discover purpose in our lives and maintain self-love, regardless of who engages in discrimination or attempts to bring us down” (Gowalla et al., 2019). The quote places responsibility on the queer individual to struggle and persevere. The quote suggests that, if a queer person were to die by suicide, that person had been unable to discover meaning in life and practice self-love. This sentiment that suicide is not a way to solve one’s problems is echoed in a quote from a female politician: “Suicides cannot be considered a solution to any problem” (TNN, 2008).

Furthermore, an activist comparing the reactions of a queer person from the past to the actions of an individual who died by suicide terms the former as better and advises queer people to “toughen up”:

Meanwhile, what really surprises all of us is why did not he reach out to any of the support groups. He could’ve easily done that, for he was tech-savvy. 10 years ago…two people…reached out to me. That boy called me from an STD booth in Nagercoil. If that happened 10 years ago, I am shocked that this boy did not reach out to anyone in this time when things are quite different, and the support groups are at your fingertips. Yes, bullying based on one’s sexual orientation is very vicious and that could lead to self-loathing. But one should toughen up. We all went through it and battled it out on our own (Sah et al., 2019).

In another report, an activist blames queer individuals for remaining in the closet, implying that, by doing so, they have no one to help them when they face challenges: “But, if they remain closet gays, nobody can help them. There’s nobody to hold their hand and wipe their tears when danger signals appear” (Chaturvedi, 2012).

In addition, a psychologist blames queer youths for their inability to share their struggles with others: “I am saddened by the fact that youngsters from [the] gay community seclude themselves and do not want to open up to us” (Deb, 2016).

It is deeply concerning that these victim-blaming statements come from sources such as queer activists and mental health practitioners. News reports should refrain from including such statements or quoting individuals who lack knowledge on these issues. A more responsible approach would be for journalists reporting on queer suicides to educate themselves about queer issues and consult experts in queer mental health. This way, reporting can be more sensitive and avoid perpetuating harmful misconceptions and victim-blaming attitudes.

### 5.7 Pessimistic and dreading tone

The media’s portrayal of queer people often reflects a sense of sadness and hopelessness (Legge, 2018; Babayeva, 2019). Consequently, queer suicide reporting is rife with hopelessness and dread. While these elements might contribute to sensational news and increased readership, they also perpetuate a negative image of India, which seems to offer no future for queer individuals.

For instance, in the following example, a transgender activist speaks out against police atrocities that led to the death of a transgender person. However, her statements paint a harsh and cruel image of the world for transgender individuals:

There is so much violence—structural and sexual violence that the community faces on an everyday basis. We have been pushed to the fringes and margins of society. We are also exploited sexually because most of our survival comes from sex work, there aren’t that many jobs for us out there. What’s more, men accept our services, but they refuse to pay. They even steal our money and hurt us. No one is ready to accept us at workplaces, public spaces or even their neighbourhoods. When will we be treated as equals? (Paul, 2016).

This quote paints men, public spaces, neighbourhoods, and workspaces in a stereotyped and dangerous light. Similarly, another report about transgender people dying due to heartbreak quotes a transgender activist as saying, “Transgenders are a deprived lot. Left out by parents, they are in constant search for love” (Ommcom News, 2016). This suggests that all parents reject their transgender children at an early age, leading to a lack of love.

Even more concerning is when mental health experts contribute to the adverse environment. In one report, a psychologist states,

Society does not accept people with such behaviour, and people often criticise them. Often, there are no opportunities for them to grow in our society. They are unsure about their identity and often get depressed as they do not see a respectful life. We need to understand that they are born with such [a] nature and accept and respect them the way they are (Singh, 2020).

The above quote highlights two incorrect generalisations: a lack of opportunity and identity confusion.

One headline in particular has the potential to cause more damage, primarily because of its bold font and prominent placement at the top of the report: “Five suicides since Jan 2021 in Kochi, transgenders seek support” (Antony, 2022). Furthermore, another report, using quotes from suicide notes as supporting evidence, exaggerates the harassment that queer people face:

Avi’s Facebook post is a searing indictment of the harassment that LGBTQI people face in India, despite the reading down of Section 377 of the IPC in September last year. “When I die, I’m going to ask God why he made me like this. I’m going to ask him to not let gay people be born in India because people in India hate gay people—they blame the family and call them all sorts of names,” it reads (Ghosh and Ozarkar, 2019).

Suicide notes, written at times of extreme mental anguish, should not be used to prove the extent of the challenges that queer individuals face.

In the United States, HIV and gay identity were once closely linked (Altman, 1982). A similar notion is reflected in the headline, “Suicide among HIV-Positive gays rampant” (Chaturvedi, 2012). However, when one reads the actual article, it becomes clear that the term “rampant” is based on only two instances of queer men contracting HIV (Chaturvedi, 2012).

In another instance, instead of merely suggesting that society is not accepting of queer people, a headline states, “Better dead than gay? Guy committed suicide after being bullied for homosexuality” (TNN, 2019b). Another headline of a similar nature reads, “LGBT community goes in a huddle as gay youngster commits suicide in the city” (Deb, 2016). The implication is that this suicide endangered the entire community. The body of the report includes other distressing statements, such as “there are many who still find death more comforting” (Deb, 2016).

Another news headline, “Is there anything gay about them?” (TNN, 2006), hints that homosexual people live miserable, unhappy lives. The text of the report reads, “So, how difficult is it for city lesbians to stay afloat in such an unsympathetic environment?” (TNN, 2006). The lead of another problematic piece of reporting reads, “Homophobic slurs have allegedly led to one more death in the country” (Sah et al., 2019). It fails to reference similar incidents in which homophobia played a role in the demise of queer individuals. In addition, it suggests that bullying is a prevalent phenomenon and, in support of that statement, quotes a queer person: “Bullying is not a one-off incident. It’s a reality for us from the community that we face 365 days” (Sah et al., 2019). Another activist in the same report says, “People from the community are being bullied everywhere” (Sah et al., 2019).

In addition to portraying a consistently pessimistic view of life for queer singles, newspapers do the same for queer couples, suggesting that their relationships are unlikely to survive societal challenges. One quote exemplifies the difficulty that queer people face in sustaining their relationships: “Society does not accept such [queer] relationships” (TNN, 2006). This broad generalisation might lead queer individuals to believe that their love lives are destined to fail. Another headline, “A lesbian couple’s suicide attempt in Mumbai is just one piece of a tragic pattern” (Dore, 2016), is accompanied by the following quote: “If incidents of young women in relationships being driven to commit suicide were to be plotted on a map and along a timeline, there would be a dot in almost each year and each state” (Dore, 2016).

Similarly, the headline “Meerut lash on lesbian couple” (The Telegraph, 2006) uses synecdoche to suggest that the entire city stands against the lesbian couple. The following two headlines also imply that queer couples are destined to experience death as their ultimate fate: “Same-sex relationships: Punished in life, death” (Piyasree Dasgupta, 2011) and “What’s the fate of same-sex lovers in times of homophobia?” (Dasgupta, 2018a).

With regard to the second headline, the report judges the fate of queer couples based on two cases of suicide pacts involving queer couples over 7 years, leading to the generalised belief that all queer relationships are doomed. However, the report quotes an activist whose words suggest that the heading is misleading: “I would not say that suicide pacts of lesbian lovers are on the rise in Bengal” (Dasgupta, 2018a). Despite this, the headline does not reflect a positive development; rather, it maintains a terrifying undertone.

Why do the news media frequently depict queer individuals in tragic situations? Sukthankar (1999, p. 23) argues that queer death, love, and elopement are sensational and scandalous. When presented with overstatements and sensational language, these portrayals can instil fear and terror in readers. Studies reveal that reporting practices that provide readers with optimism and assistance in the face of a mental breakdown receive little attention (Kar et al., 2022b, p. 4; Raj et al., 2022, p. 8). On the other hand, sensational language tends to draw more attention (Raj et al., 2022, p. 8). As a result, the news media often adopt pessimistic and dread-filled tones and maintain a limited focus on hope and helpful material, which should be more prominent.

It is important to consider the fact that readers of suicide reports might include impressionable queer youth. Therefore, journalists should avoid portraying India as an inhabitable place for them. Such portrayals might make queer youth susceptible to suicide by causing them to believe that there is no place for them in the world. Unfortunately, some newspapers perpetuate this harmful representation. To address this issue, the media should refrain from exaggeration and instead provide helpful information to address mental health issues.

However, the continuous juxtaposition of suicide, sadness, and queerness can result in these incidents being stereotyped. Thus, it means that suicide is associated with queerness in some way when young people are concerned, as it seems like these are the correct actions for a queer child or adolescent to behave normally in society (Cover, 2012a, p. 12). To compound matters further, portraying queer suicide negatively only serves to amplify the already present stigma and marginalisation towards queer populations.

The reason is that it ultimately justifies hatred against queer people and the inequality they face on a daily basis. If we believe they are not fully human, then why would they deserve rights as humans? Instead of this approach, news stories need to be presented with direct facts, without any kind of sensationalism, and a journalist should retain impartiality rather than a passionate tone (Ramadas et al., 2014, p. 4; Jain and Kumar, 2016, p. 3).

On the other hand, the tragic tone of a narrative might draw attention from indifferent policymakers and encourage the system to take action. Additionally, it might cajole empathetic readers into being more accepting of queer people. However, it is essential to consider the impact that these reports have on young queer individuals who read them. To strike a balance, news reports must highlight the issues while avoiding creating a perception that queer individuals cannot lead happy lives. Reporters can infuse positivity into their stories about suicide by including survivors’ stories and conveying the hopeful message that mental health challenges can be overcome.

## 6 Conclusion

Despite the long period (2005–2022) of study, the frames emerging from the investigated suicide stories have shown little to no significant change. The consistent use of substandard, sensationalist, and stereotypical portrayals of queer people in the media serves to further marginalise and criminalise them, inadvertently causing harm.

Homosexuality was previously a taboo topic; now, reporting on queer individuals has become a means for media outlets to gain readership (Ghosh, 2022, p. 2). Unfortunately, this journey from invisibility to public exposure has resulted in one form of marginalisation being replaced by another. World Health Organization (2014) advocates for a sympathetic representation of suicide in media as a crucial suicide prevention tactic. By identifying insensitive media frames in queer suicide reporting, this research encourages responsible and sensitive media portrayals, ultimately contributing to better mental health outcomes for queer individuals.

From our analysis of Hindi and English news reports on queer suicide cases, several concerning patterns have emerged:

1. Reports often categorise queer couples into gender binary lines based on their appearance and behaviour, portraying them as traditional heterosexual couples. This perpetuates the assumption that queer relationships must conform to societal norms and reinforces harmful stereotypes.
2. News reports frequently delve into explicit details of the couple’s sexual behaviour, portraying them as reckless and intoxicated by queer love. This sensationalist approach violates their privacy and depicts them as sexual predators deserving of a deadly fate, thereby exacerbating the stigma against queer individuals. It shifts the blame away from the society and heteronormativity that have actually compelled these individuals to resort to such extreme measures.
3. Family members refuse to accept their children’s identities and offer various explanations to journalists. They claim that their children were simply close friends of the deceased, assert that their children were coerced into queer relations by their partners, or state that their children were straight allies, not queer. This refusal to acknowledge a child’s true identity implies that parents still view queerness as something that might bring shame upon the deceased individual and their family members.
4. The reporting exhibits a dread-filled and pessimistic tone, depicting queer people as leading unhappy and tragic lives from which death is the only escape. Such portrayals can have a detrimental impact on queer youth, instilling a sense of hopelessness and disinterest in living.
5. News reports tend to cite queer relationships as the primary cause of suicide among queer individuals, overlooking the role of queerphobia and heteronormativity. This implies that being straight is the only path to happiness, further marginalising queer individuals.

These findings underscore the need to educate journalists on sensitively reporting queer suicide cases. A thorough examination of the current journalism course curriculum is necessary to determine if it adequately addresses reporting on news related to the queer community. Any deficiencies in the curriculum should be promptly addressed to equip aspiring journalists with the skills needed to handle queer suicide cases appropriately and with sensitivity. Media organisations must provide training to journalists specifically on reporting queer suicides.

Instances of poor reporting passing editorial checks and being published on news portals without triggering alarms among editors highlight a failure in editors’ performance, often attributed to a lack of training or a focus on maximising clicks. This emphasises the need for enhanced editorial oversight and training to ensure responsible reporting on sensitive topics involving the queer community.

Due to the sub-part quality of reporting on queer individuals in India, the LIKHO initiative was established in 2015. Its primary goal is “to encourage sensitive and erudite coverage of LGBTQ issues in the Indian media” (About Likho, 2024). Similarly, in 2022, the independent collaborative journalism project queerbeat was launched to set a precedent for Indian media outlets. Queerbeat seeks to tackle the persistent problems of underreporting and misreporting concerning the identities, stories, and issues of queer individuals, pointing out that mainstream media have frequently overlooked and dehumanised this community (About Queerbeat, 2024). In alignment with this mission, The News Minute and Queer Chennai Chronicles introduced the Inqlusive Newsrooms: LGBTQIA+ Media Reference Guide (Queer Chennai Chronicles and The News Minute, 2023). These initiatives foster optimism regarding a potential enhancement in the quality of queer suicide coverage in both English and regional language media in India.

It should be noted that this study’s focus on content analysis limited to Hindi and English newspapers overlooks the vast linguistic diversity of India, encompassing 22 major languages recognised by the Indian constitution (Language Education, 2021). Future research should expand its scope to include other regional languages and thereby comprehensively assess the quality of queer suicide reporting across the nation.

Furthermore, while this research concentrates on written digital print media, there remains a significant gap in understanding the quality of news reporting on queer issues in broadcast media. Separate investigations should be undertaken to evaluate the portrayal of queer individuals in television and radio broadcasts.

Additionally, the absence of interviews with journalists in this study is a limitation. Interviews could offer valuable insights into the current state of journalism education and training regarding queer reporting, as well as identify shortcomings or biases within media practices. The integration of interviews with journalists into future research endeavours would enrich our understanding of the challenges and opportunities involved in enhancing media representation of the queer community in India.

## Data availability statement

The original contributions presented in the study are included in the article/supplementary material, further inquiries can be directed to the corresponding author.

## Author contributions

KBJ: Conceptualisation, Data curation, Formal analysis, Funding acquisition, Investigation, Methodology, Project administration, Resources, Software, Supervision, Validation, Visualisation, Writing – original draft, Writing – review & editing.

## Footnotes

^1^In this context, the umbrella term “queer” refers to a sexual and gender minority individual. This categorisation is necessary due to unclear self-identity or incorrect identification mentioned in the reports of deceased individuals.

^2^Copycat suicide involves individuals imitating suicidal behaviour after learning about another person’s suicide.

^3^This quote, along with the subsequent quotes presented without inverted commas, represents my own translation of news reports originally written in the Hindi language.

^4^This frame is closely related to the abhorrent and frenzied queer love news frame. However, the distinction lies in the fact that the latter explains how immoral love leads to suicide, while the former only describes the negative aspects of love and its harmful influence on others.

^5^Here, based on the reporters’ limited account, we have identified a potential factor. It must be noted that suicide is caused by several factors and cannot be attributed to a single cause.

^6^For another example of police negligence towards queer suicides, consider this excerpt from a report: “The police intend to conclude the case, classifying it as a suicide. ‘Since both of them have died, what is left to investigate for us?’ Yadav said” (Roy, 2020).
